# Identification of a novel compound heterozygous *SMG9* variants in a Chinese family with heart and brain malformation syndrome using whole exome sequencing

**DOI:** 10.1186/s12920-022-01217-9

**Published:** 2022-03-23

**Authors:** Qi Yang, Zailong Qin, Qinle Zhang, Shang Yi, Sheng Yi, Jingsi Luo

**Affiliations:** 1 Guangxi Key Laboratory of Birth Defects Research and Prevention, Guangxi Key Laboratory of Reproductive Health and Birth Defects Prevention, Maternal and Child Health Hospital of Guangxi Zhuang Autonomous Region, No. 59, Xiangzhu Road, Nanning, China; 2 Department of Genetic and Metabolic Central Laboratory, Maternal and Child Health Hospital of Guangxi Zhuang Autonomous Region, Nanning, China

**Keywords:** SMG9-deficiency syndrome, SMG9, Whole exome sequencing, Novel variants

## Abstract

SMG9-deficiency syndrome, also known as heart and brain malformation syndrome, is a very rare congenital genetic disorder mainly characterized by brain, heart, and growth and developmental abnormalities. This syndrome is an autosomal recessive disease resulting from mutations in the *SMG9* gene, which encodes a critical component of nonsense-mediated mRNA decay. Thus far, only twelve SMG9 deficiency patients have been reported with five novel homozygous *SMG9* mutations. The most frequent characteristic features of these patients are facial dysmorphism, severe global developmental delay, intellectual disability, congenital heart disease, growth restriction, microcephaly, and brain abnormalities. Herein, whole exome sequencing was performed to identify novel compound heterozygous *SMG9* variants (NM_019108.3: c.1318_1319delAG (p.Ser440*) and c.947A>G (p.His316Arg)) in the proband, who exhibited syndromic intellectual disability. Mutations were confirmed as segregating in his affected sister and other unaffected family members by Sanger sequencing. The patients we describe here have a similar dysmorphology profile associated with SMG9-deficiency syndrome. Comparing the phenotype with that of patients in published reports, our patients can walk independently and their growth parameters are normal. In addition, short stature, failure to thrive, and microcephaly were not observed. Possible residual function of the H316R *SMG9* variant could explain the milder phenotype observed in our patients. Our report is the first description of a non-consanguineous Chinese pedigree with novel compound heterozygous variants in the SMG9 gene. The molecular confirmation of the patient expands the genetic spectrum of SMG9-deficiency syndrome, and the manifestation of SMG9-deficiency syndrome in the patient provides additional clinical information regarding this syndrome.

## Introduction

The *SMG9* gene, located at 19q13.31, encodes a regulatory subunit of the SURF complex, which is a translation termination complex during nonsense-mediated decay (NMD) [1]. NMD is a conserved posttranscriptional surveillance pathway that reduces the production of harmful truncated proteins translated from transcripts with premature stop codons (PTC) to ensure the fidelity and accuracy of the process from the transcription of genetic information to protein synthesis [2–5]. It plays an important role in many biological processes, including embryonic development, cell differentiation, stress responses and immune response [6]. Disruption of NMD can lead to a plethora of human genetic diseases [7]. As a member of the core factors involved in NMD, SMG9 supports and stabilizes the formation of the SMG1 complex (SMG1C) by binding to SMG1 and SMG8, and it is involved in PTC recognition, which is a key step in the degradation of mRNA containing PTC [1, 8]. The inability of cells to recognize transcripts containing PTCs with SMG9 loss-of-function mutations further indicates that SMG9 plays a key role in post-transcriptional regulation and monitoring [9]. SMG9 is highly conserved from archaea to eukaryotes and is expressed widely across many tissue types in humans. This implied that functionally impaired SMG9 would lead to a severe disease phenotype.

**Fig. 1:**
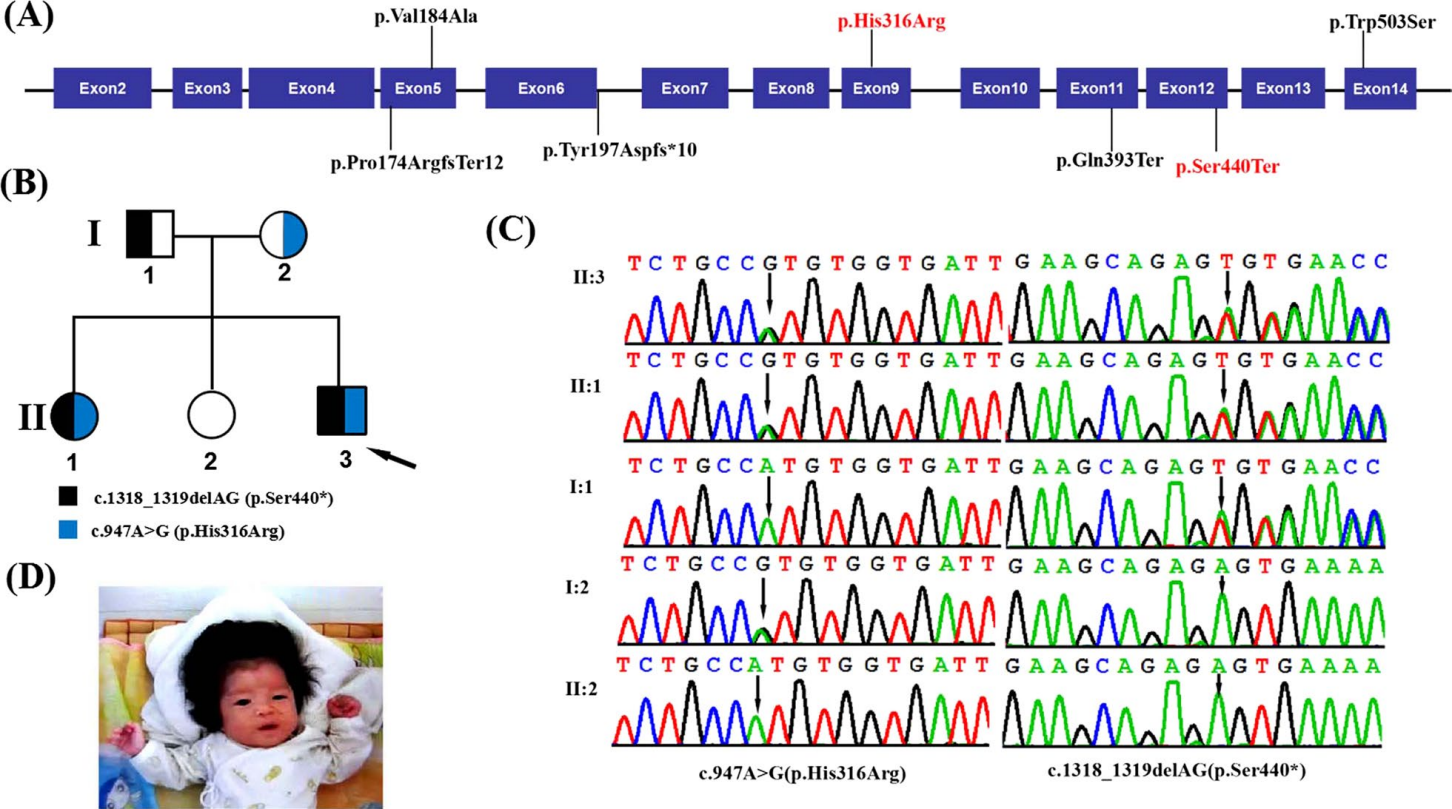
Clinical and genetic features. **A** The distribution of all variants detected so far in fourteen patients with *SMG9* variants. **B** Pedigree chart of the family of the patients with SMG9-deficiency syndrome. The proband is indicated by a black arrow. **C** Sanger sequencing DNA chromatograms of *SMG9* indicating the frameshift c.1318_1319delAG(p.Ser440*) variant inherited from the mother and the missense variant c.947A>G (p.His316Arg) was transmitted by the father. **D** Facial clinical features at the age of 4 months. Note the presence of prominent metopic suture with broad nasal bridge, low set malformed ears and left-sided ptosis

Homozygous loss-of-function variants in the *SMG9* gene (OMIM: 613176) were recently described to cause a neurodevelopmental disorder characterized by intellectual disability and multiple malformations in twelve affected individuals from worldwide [9–13] (Fig. 1A). The most frequent manifestations of these subjects with mutations in the *SMG9* gene are microcephaly, cerebral malformations, intellectual disability, congenital heart defects, and ocular anomalies. However, the severity of the phenotype caused by *SMG9* mutations remains to be fully explored. Additional reports on *SMG9* gene mutations and their phenotypes will therefore be essential to the understanding of this condition. Herein, we report compound heterozygous *SMG9* (NM_019108.3) variants, c.947A>G (p.His316Arg) and c.1318_1319delAG (p.Ser440fs), identified in a Chinese family with two affected individuals, and we describe the patient’s associated clinical profile.

## Materials and methods

### Ethical compliance

A Chinese family was recruited form the Maternal and Child Health Hospital of Guangxi Zhuang Autonomous Region with a total of five members, with two affected patients and three unaffected individuals (Fig. 1B). The study was approved by the Institutional Review Board and Ethics Committee of Guangxi Maternal and Child Health Hospital, and detailed written informed consent was obtained from the patients’ parents.

### Whole-exome sequencing and Sanger sequencing

Peripheral anticoagulated whole blood samples (2 ml) were collected from all family members. Genomic DNA was extracted with a commercial DNA extraction kit (Zeesan Biotech Co., Ltd, Xiamen, China). To identify the potential pathogenic variants in the patients of the family, whole-exome sequencing (WES) was performed using genomic DNA of the proband. Exome enrichment was performed using a commercial capture kit (Sure Select Human All Exon, v5, Agilent Technologies, Santa Clara, CA, USA). The enriched library was sequenced on the Illumina HiSeq 2000 platform (Illumina Biotechnology, San Diego, CA, USA). The GRCh37/hg19 reference genome was aligned with the reads obtained using the BWA Multi-Vision software package (v. 0.7.15). Variant calling was performed with the Genome Analysis Toolkit (GATK) and the variant annotation with TGex software (LifeMap Sciences, Alameda, CA).

SIFT (http://sift.jcvi.org/), PolyPhen2 (http://genetics.bwh.harvard.edu/pph2/), CADD (https://cadd.gs.washington.edu/snv), and MutationTaster (http://www.mutationtaster.org/) were used to predict the effects of variants on protein structure and function. A 3D model of the SMG9 protein was constructed using SWISS-MODEL (https://swissmodel.expasy.org/). Co-segregation analysis of *SMG9* variants were performed with Sanger sequencing among family members (Fig. 1C). For validation PCR, *SMG9* forward 5′-CCTGATTAGTCTGGGCAGAAG-3′ and reverse 5′-AGGATCCCTCTGGCTGCT-3′ primers were used for exon 9 amplification, and forward 5′-CTCCTGATCCTGCTTTGACTG-3′ and reverse 5′-TTGTCTCTCCATGAACCTGTTG-3′ primers were used for exon 12 amplification. The pathogenicity of the variants was classified according to ACMG/AMP guidelines [14].

## Results

### Clinical phenotype

The proband (II:1), a 7-year-old male, was the third child of physically healthy non-consanguineous parents (Fig. 1B). He was born at full term with normal measurements (51.2 cm; 3340 g). He was admitted to the Pediatric Endocrine Guangxi Zhuang Autonomous Region Women and Children Care Hospital due to severe intellectual disability and gait disturbance when he was 5 years old. He started to sit unsupported at 11 months and walked at 31 months, but continues to exhibit an unsteady gait. He currently has no meaningful language. He had mild facial dysmorphic features including a broad nasal bridge, low set malformed ears, and left-sided ptosis (Fig. 1D). He has Duane syndrome. He also suffered from congenital heart disease consisting of a repaired atrial septal defect and a ventricular septal defect. According to the Wechsler Intelligence Scale for Children at the age of 5, his Full Scale IQ was 45. He has recurrent stereotypical body rocking, hand flapping, and spinning. Brain magnetic resonance imaging at 4 years showed mild generalized brain atrophy. He does not have hearing problems. Growth parameters were also within the normal range (At 5 years of age: Height, 110.3 cm, 50%, 0.0SD; weight, 20.2 kg, + 0.4SD; head circumference, 50 cm, − 0.5SD). His karyotype was 46, XY. His 10-year-old sister was also found to have a similar phenotype in the form of severe psychomotor delay, mild craniofacial dysmorphism, and congenital heart defects. Full clinical details for each patient are shown in Table 1.

**Table 1:** Comparison of the clinical phenotype of patients with *SMG9* mutation reported by other groups

Patients clinical data	Our patient		Altuwaijri et al. [12]		Lemire et al. [11]	Shaheen et al. [9]			Lecoquierre et al. [10]	Rahikkala et al. ([13], 5 patients)
	Proband	Affected sister	Patient 1	Patient 2		Family I	Family II-proband	Family II-proband's first cousin		
Variants in SMG9 (NM_019108.3)	c.947A>G (p.His316Arg) and c.1318_1319delAG (p.Ser440*)		c.701+4A>G (p.Tyr197Aspfs*10)		c.1508G>C (p.Trp503Ser)	c.520_521delCC (p.Pro174Argfs*12)	c.701+4A>G (p.Tyr197Aspfs*10)		c.1177C>T (p.Gln393*)	c.551T>C (p.Val184Ala)
Gender	Male	Female	Male	Female	Female	Female	Female	Female	Female	Male
Age at last examination	5 year	10 years	Died at 25 months	Died at 1 h after birth	7 years	Died at 7 weeks	Died at 7 weeks	8 years	5 years	≥ 25 years in all the 5 patients
Neurodevelopment	Severe ID	Severe ID	Global developmental delay	NA	Severe ID	NA	Global developmental delay	Global developmental delay	Severe psychomotor developmental delay	1/5 mild ID, 3/5 moderate ID, 1/5 borderline mild/moderate ID
Height, weight, head circumference	110.3 cm (50%, 0.0 SD), 20.2 kg (+ 0.4SD), 50 cm (− 0.5SD)	132.1 cm (10%, − 1.1SD), 31.2 kg (49.5%, 0.0SD)	74 cm (< 1%, − 3.7SD), 8.9 kg (< 1%, − 3.1SD), 44 cm (< 1%, − 3.2SD)	NA	118.5 cm (30%), 25.1 kg (75%), 49.5 cm (25%)	44 cm (− 2.4SD), 2.130 kg (5%), 31.5 cm (− 2.2SD)	47 cm (15%, − 2.2SD), 2.26 kg, 32 cm (− 1.8SD)	69 cm (− 4.8SD), 6.3 kg (− 5.2SD), 39 cm (− 6SD)	All three metrics were less than 3SD at the age of 2.5 years	3/5 short stature, 2/5 microcephaly
Age of walking	31 months but continues to exhibit an unsteady gait	35 months but continues to exhibit an unsteady gait	NA	NA	7 years but walk with a walker and ankle foot orthosis	NA	NA	Unstable to sit alone	Inability to walk	≤ 20 month
Development of speech and language skills	No meaningful language	Nonverbal	NA	NA	Nonverbal	NA	NA	Nonverbal	Nonverbal	Markedly delayed
Behavioral anomalies	Recurrent stereotypical body rocking, hand flapping and spinning	Recurrent hand flapping		NA	Recurrent hand flapping and stereotypical body rocking					
Brain radiologic features	Mild generalized brain atrophy hypomyelination, and small globus pallidus and putamen	Not performed	Brain atrophy, abnormally shaped lateral ventricles, thin corpus callosum, cerebellar and pontine atrophy with flattening of the pons ventral aspect, diffuse	Not performed	Normal	Dandy–Walker malformation	Brain atrophy, decreased myelination, and Dandy–Walker malformation	Generalized brain atrophy, prominent ventricular system and thin corpus callosum	Enlarged fourth ventricle with dysmorphism, a parieto-occipital junctional stroke, thin corpus callosum, a cavum septum pellucidum, and a Blake's pouch cyst	1/5 abnormal 2/5 normal
Feeding difficulty	Yes	Yes	Yes	NA	Yes				Yes	Yes
Muscular hypotonia	Yes	Yes	Yes	NA	Yes	NA	Yes	Yes	Yes	2/5 abnormal
Ocular anomalies	Duane syndrome	Poor vision	Bilateral sutural cataract	NA	Bilateral Duane syndrome type III, high hyperopia, and astigmatism	Microphthalmia	Microphthalmia		Hypertelorism, ptosis, and bilateral cataracts	4/5 abnormal
Cardiovascular	Atrial septal defect and VSD	VSD, hypoplastic tricuspid valve	Interrupted inferior vena cava, a small ASD with left to right shunt	Ectopia cordis	ASD and a large perimembranous VSD	Interrupted aortic arch, hypoplastic tricuspid and aortic valves, large muscular VSD	Large VSD	VSD	VSD, aortic valve bicuspidy and aortic dilatation	1/5 abnormal 4/5 normal
Infections	No	No	Yes	NA	Yes	Yes			Yes	Yes
Facial dysmorphic fetaures	Broad nasal bridge, low set malformed ears and left-sided ptosis, prominent forehead	Broad nasal bridge, low set malformed ears, prominent forehead	Microretrognathia, anteverted nares, and hypertelorism, unilateral ear malformation and bilateral external auditory canal stenosis	NA	Long and oval face, narrow mouth, bifrontal narrowing with frontal bossing, prominent nose with bulbous tip, and high anterior hairline	Prominent forehead and occiput, low set malformed ears, wide anterior fontanelle, depressed nasal bridge and anteverted nares, high arched palate	Narrow forehead, prominent metopic suture, posteriorly rotated ears with attached lobules, hypertelorism, small eyes, broad nasal bridge, full and everted lower lip, right-sided cleft lip	Dysmorphic features	Left cleft lip and palate, transient frontal white hair lock, bi-temporal retraction with frontal bossing, upslanting palpebral fissures with hypertelorism, broad nasal bridge, midface hypoplasia, low-set posteriorly rotated ears with attached lobules, Widow's peak	Abnormal

*ID* intellectual disability, *SD* standard deviation from the mean, *ASD* atrial septal defect, *VSD* ventricular septal defect, *NA* not available

### Mutation analysis

WES was performed on the proband, and sequencing reads of 5.4 Gbp were generated. A total of > 99% of the targeted regions were covered with a depth of more than 10×. A total of 26,201 SNV or indel variants were identified in coding regions and splice sites (splicing junction 10 bp). After removing synonymous variants, and removing the variants with a minor allele frequency (MAF) > 1% in gnomAD, ESP, 1000G and our internal database, there were 1253 variants remaining with a MAF < 0.01. Furthermore, according to the ClinVar databas, the neutral, likely benign and benign variants were also excluded. Clinical features included intellectual disability, gait disturbance, motor delay, dysphasia, broad nasal bridge, low set malformed ears, left-sided ptosis, duane anomaly, septal defect, ventricular septal defect, stereotypical body rocking, and brain atrophy were regarded as filtration parameters for variant screening. Using TGex software (LifeMap Sciences, United States), nine candidate variants matched with known phenotypes in eight genes (*SMG9*, *NIPBL*, *ATN1*, *NPC1*, *MED23*, *METTL23*, *RBM12*, *DEPDC5*) were subsequently extracted. Two heterozygous *SMG9* variants, c.947A>G (p.His316Arg) and c.1318_1319delAG (p.Ser440*), were identified in the proband (Fig. 1C). Sanger sequencing further revealed that the heterozygous c.947A>G (p.His316Arg) and c.1318_1319delAG (p.Ser440*) variants were identified in the father and mother, respectively, and that his affected sister (II-1) also had these variants (Fig. 1C). However, another sister (II-2) was unaffected, and Sanger sequencing showed she did not have either variant.

**Fig. 2:**
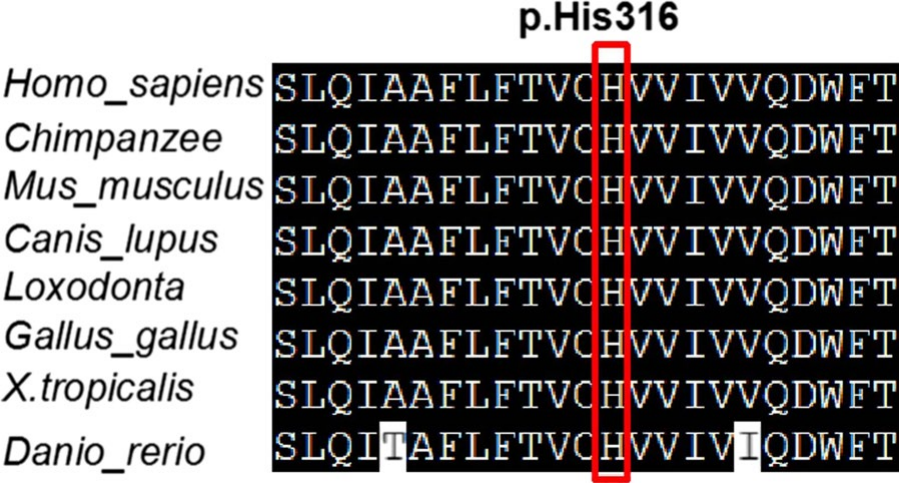
Multispecies alignment showing the strong conservation of SMG9 p.His316

**Fig. 3:**
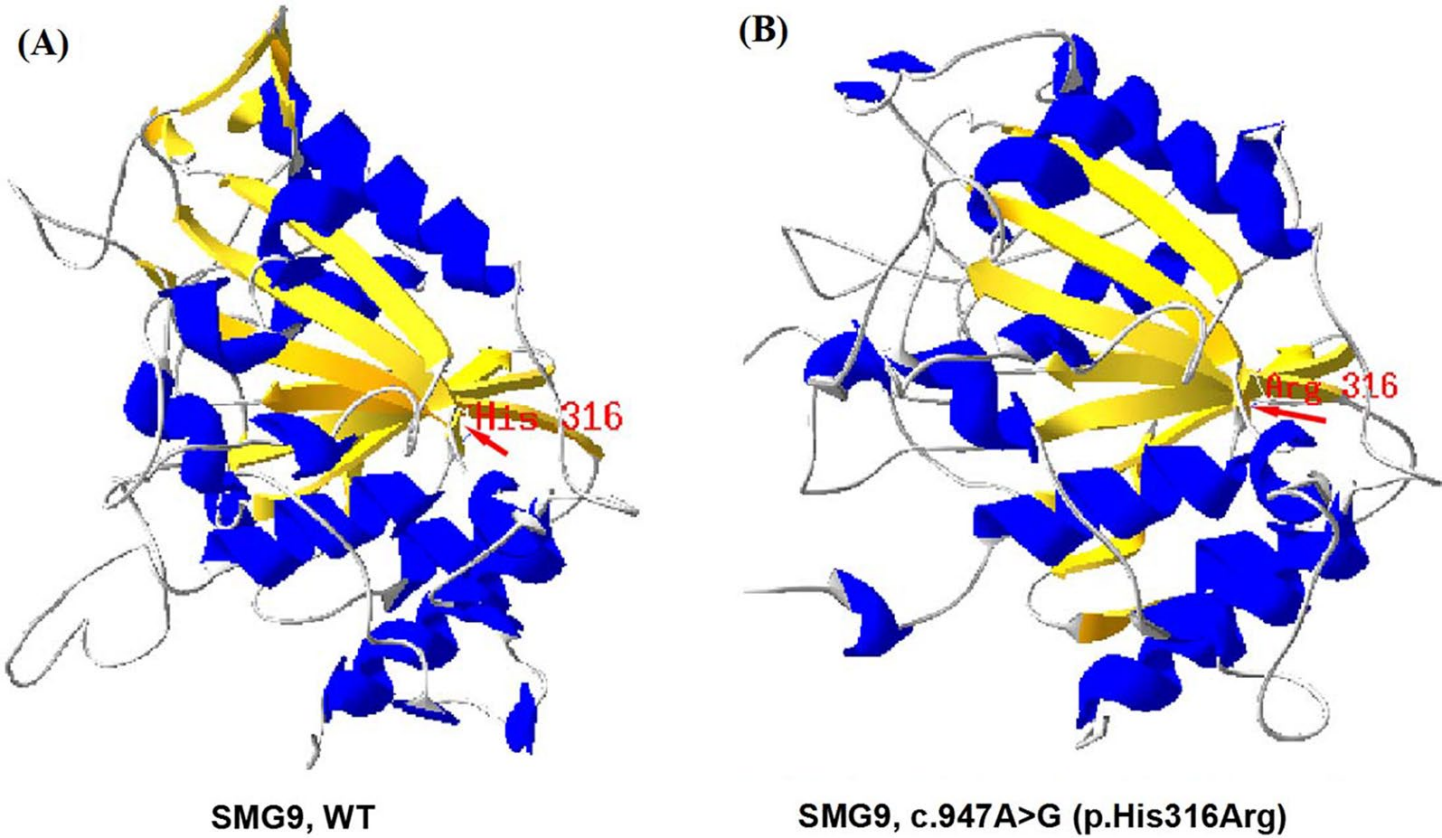
**A**, **B** Three-dimensional structures of SMG9 protein. **A** Wild-type, **B** c.947A>G (p.His316Arg) mutant-type. The arrows indicated the location of p.His316

The variant c.1318_1319delAG(p.Ser440*) was absent in the Human Gene Mutation Database (http://www.hgmd.cf.ac.uk/ac/), HPSD (http://liweilab.genetics.ac.cn/HPSD/), dbSNP (http://www.ncbi.nlm.nih.gov/SNP/), ExAC, and gnomAD (https://gnomad.broadinstitute.org/). It was located in the twelfth exon of the *SMG9* gene and causes a premature termination codon, leading to a loss of function. The functional prediction for c.1318_1319delAG(p.Ser440*) was disease-causing by MutationTaster. The other *SMG9* variant c.947A>G (p.His316Arg) is present in the Genome Aggregation Database (gnomAD v.2.1.1), with a minor allele frequency of 0.00000398. The variant c.947A>G (p.His316Arg) is located in the ninth exon of the *SMG9* gene and in the nucleotide-binding G-fold domain of the SMG9 protein, which is required for the interaction between SMG9 and the G-like domain of SMG9 [15–17]. Multiple sequence alignment revealed that the sequence at residue 316 is highly conserved in different organisms (Fig. 2). The variant c.947A>G (p.His316Arg) was predicted to be deleterious by SIFT, PolyPhen2, and CADD. The Software SWISS-MODEL was used to predict the 3D structures of the wild type (WT) and mutant SMG9 (Fig. 3) protein. 3D modeling of the WT and mutated protein sequences indicated that for the SMG9-H316R variant, the additional arginine gained from the variant changes the secondary and tertiary structures by reducing a local α-helix. According to the AMP/ACMG guidelines for the interpretation of sequence variants [6], c.947A>G (p.His316Arg) was assessed to be likely pathogenic (PM1, PM2_supporting, PM3, PP3, PP1_supporting), and c.1318_1319delAG(p.Ser440*) was assessed to be pathogenic (PVS1, PM2_supporting, PP1_supporting).

### Genotype–phenotype correlations

To date, a total of 12 patients with pathogenic *SMG9* variants have been reported in the literature [9–13]. Clinical and molecular features of the 12 patients and of our patients are summarized in Table 1. By extensive literature analysis, we compared the phenotypes of 6 patients with homozygous missense variants and 6 patients with loss-of-function variants (LoF; including frameshifts, nonsense variants, and splice sites). Of the 6 patients with homozygous missense variants, some milder phenotypes were observed, including independent walking (5/6), normal speech (5/6), mild to moderate intellectual disability (5/6), and normal growth (6/6). We also noticed that only one of these patients had a brain malformation, and two had congenital heart disease. While in patients with homozygous loss-of-function mutations, severe intellectual disability (4/4), inability to walk (3/3), ventricular septal defect (6/6), growth Restriction (3/3), microcephaly (5/5), and brain abnormalities (5/5) were observed.

## Discussion

Heart and brain malformation syndrome is a rare neurodevelopmental disorder. In 2016, Shaheen et al. reported for the first time that *SMG9* mutations were found in three children with brain and heart malformations from two unrelated families through WES [9]. Lemire et al. further expanded the disease phenotype by studying *SMG9* mutation in a patient with intellectual disability and multiple malformations [11]. To date, only twelve patients with SMG9-deficiency syndrome have been reported [9–13]. The most frequent characteristic features of these subjects with SMG9-deficiency are facial dysmorphism, congenital heart defects, severe intellectual disability, growth restriction, microcephaly and brain abnormalities. In the current study, we performed WES and identified compound heterozygous variants in the *SMG9* gene in a Chinese family that included two patients. The patients showed the common phenotypes associated with SMG9-deficiency syndrome, including facial dysmorphism, a degree of intellectual disability, developmental delay, mild generalized brain atrophy, and congenital heart defects. Therefore, the patients were diagnosed with heart and brain malformation syndrome. Regarding developmental delay, our patients could walk independently and had normal growth. In addition, short stature, failure to thrive, and microcephaly were not observed in our patients.

In the patients described in the present study, the biallelic *SMG9* variants identified were a combination of frameshift and missense variants. The c.1318_1319delAG (p.Ser440*) variant was a novel frameshift variant located in the ninth exon of the SMG9 gene. It may act similarly to other loss-of-function variants (LoF; including frameshifts, nonsense variants, and splice sites) of *SMG9*, like c.701+4A>G and c.520_521delCC(p.Pro174Argfs*12) that have previously been reported [9]. These variants result in no protein production with a significant decrease in mRNA level due to NMD degradation [9]. The other variant, c.947A>G (p.His316Arg), is located in the G-fold domain. This domain is involved in the formation of SMG8-SMG9 heterodimers and could impact the kinase activity of SMG1 [15]. The protein 3D structural analysis of SMG9-H316R suggests that the mutation leads to a decreased number of α-helices and disruption of the integrity of the G-fold domain. The variant is predicted to affect the formation of the SMG1-SMG8-SMG9 complex, altering the kinase activity of SMG1 [15–17]. According to the ACMG/AMP standards and guidelines [12], the novel c.1318_1319delAG (p.Ser440*) variant is pathogenic according to the PVS1, PM2_supporting, and PP1_supporting criteria, while the novel c.947A>G (p.His316Arg) variant is likely pathogenic according the PM1, PM2_supporting, PM3, PP1_supporting, and PP3 criteria.

To date, only twelve affected individuals have been reported with homozygous variants in *SMG9*, including two missense variant, one nonsense variant, one frameshift variant, and one splicing variant [9–11]. Clinical and molecular features of the 12 patients and of our patients are summarized in Table 1. Of note, patients with *SMG9* homozygous missense variants exhibited a milder phenotype [11, 13], while patients with homozygous loss-of-function variants (LoF; including frameshifts, nonsense variants, and splice sites) exhibited a more severe phenotype [9, 10, 12]. This suggests the degree of phenotypic defects is dependent on the variable degree of functional SMG9 impairment. In the current study, the patient presented with somewhat milder phenotype and was identified to carry both a frameshift and a missense variant. It appears that p.Ser440* is associated with a similar LOF variant to the *SMG9* variants reported, while the p.His316Arg missense variant may result in partial loss-of-function of the SMG9 protein. Possible residual function of the H316R SMG9-carrying protein could explain the milder phenotype observed in our patients. These results are limited by the currently reported cases and variants, and as the number of patients increases, further refinement of the phenotype and identification of genotypic effects and other phenotypic determinants are expected. Further functional studies of these variants are needed to enhance our understanding of the disease and its mechanisms of action.

The underlying mechanism of SMG9 causing the congenital syndrome with multisystem abnormalities still remains to be elucidated. Previous study revealed that *SMG9* deletion plays an established role in NMD, but there is no evidence that NMD in SMG9 deficiency causes widespread interference with the degradation of transcripts containing PTC [9]. In addition, PTC-containing transcripts undergo efficient degradation in the context of SMG9 deficiency, and the *SMG9* mutant transcript itself is also regulated by NMD in the cells of affected individuals [9, 18]. Although the severity of the phenotype of individuals with different variant types is variable, the global homogeneity of the phenotype among all affected individuals suggests that the disease may be caused by a consistent dysregulated mechanism. Thus, SMG9 may have other unknown functions besides NMD that contribute to the pathogenic mechanism of SMG9-related syndromes.

## Conclusions

In summary, we identified a novel compound heterozygous variant in the *SMG9* gene in two patients from the same family with a degree of intellectual disability, developmental delay, and other congenital abnormalities. This is the first description of a non-consanguineous Chinese pedigree with *SMG9* variants. These variants were associated with a milder phenotype of SMG9-deficeny syndrome, which provides new insights into phenotypes caused by different variant combinations. The molecular confirmation of these SMG9-deficiency syndrome patients expands the clinical profile of patients with SMG9-deficiency syndrome as well as the SMG9-deficiency syndrome-associated *SMG9* variant spectrum.

## Data Availability

The datasets analyzed during the current study have uploaded the associated datasets of this study to the SRA—NCBI repository, the Sequence Read Archive (SRA) accession number is: PRJNA798084.

## Abbreviations

NMD: Nonsensemediated mRNA decay
PTC: Premature stop codons
SMG1C: SMG1 complex
WES: Whole-exome sequencing
GATK: Genome Analysis Toolkit
WT: Wild type

## References

[1] Yamashita A, Izumi N, Kashima I, Ohnishi T, Saari B, Katsuhata Y, Muramatsu R, Morita T, Iwamatsu A, Hachiya T (2009). SMG-8 and SMG-9, two novel subunits of the SMG-1 complex, regulate remodeling of the mRNA surveillance complex during nonsense-mediated mRNA decay. Genes Dev.

[2] Maquat LE (1995). When cells stop making sense: effects of nonsense codons on RNA metabolism in vertebrate cells. RNA.

[3] Popp MW, Maquat LE (2013). Organizing principles of mammalian nonsense-mediated mRNA decay. Annu Rev Genet.

[4] Lykke-Andersen J, Bennett EJ (2014). Protecting the proteome: eukaryotic cotranslational quality control pathways. J Cell Biol.

[5] Karousis ED, Nasif S, Mühlemann O (2016). Nonsense-mediated mRNA decay: novel mechanistic insights and biological impact. Wiley Interdiscip Rev RNA.

[6] Nasif S, Contu L, Mühlemann O (2018). Beyond quality control: the role of nonsense-mediated mRNA decay (NMD) in regulating gene expression. Semin Cell Dev Biol.

[7] Lindeboom RGH, Vermeulen M, Lehner B, Supek F (2019). The impact of nonsense-mediated mRNA decay on genetic disease, gene editing and cancer immunotherapy. Nat Genet.

[8] Deniaud A, Karuppasamy M, Bock T, Masiulis S, Huard K, Garzoni F, Kerschgens K, Hentze MW, Kulozik AE, Beck M (2015). A network of SMG-8, SMG-9 and SMG-1 C-terminal insertion domain regulates UPF1 substrate recruitment and phosphorylation. Nucleic Acids Res.

[9] Shaheen R, Anazi S, Ben-Omran T, Seidahmed MZ, Caddle LB, Palmer K, Ali R, Alshidi T, Hagos S, Goodwin L (2016). Mutations in SMG9, encoding an essential component of nonsense-mediated decay machinery, cause a multiple congenital anomaly syndrome in humans and mice. Am J Hum Genet.

[10] Lecoquierre F, Bonnevalle A, Chadie A, Gayet C, Dumant-Forest C, Renaux-Petel M, Leca JB, Hazelzet T, Brasseur-Daudruy M, Louillet F (2019). Confirmation and further delineation of the SMG9-deficiency syndrome, a rare and severe developmental disorder. Am J Med Genet A.

[11] Lemire G, MacDonald SK, Boycott KM (2020). SMG9-deficiency syndrome caused by a homozygous missense variant: expanding the genotypic and phenotypic spectrum of this developmental disorder. Am J Med Genet A.

[12] Altuwaijri N, Abdelbaky M, Alhashem A, Alrakaf M, Hashem M, Alzahrani F (2021). Further delineation of SMG9-related heart and brain malformation syndrome. Am J Med Genet A.

[13] Rahikkala E, Urpa L, Ghimire B, Topa H, Kurki MI (2022). A novel variant in SMG9 causes intellectual disability, confirming a role for nonsense-mediated decay components in neurocognitive development. Eur J Hum Genet.

[14] Richards S, Aziz N, Bale S, Bick D, Das S, Gastier-Foster J (2015). Standards and guidelines for the interpretation of sequence variants: a joint consensus recommendation of the American College of Medical Genetics and Genomics and the Association for Molecular Pathology. Genet Med.

[15] Li L, Lingaraju M, Basquin C, Basquin J, Conti E (2017). Structure of a SMG8-SMG9 complex identifies a G-domain heterodimer in the NMD effector proteins. RNA.

[16] Gat Y, Schuller JM, Lingaraju M, Weyher E, Bonneau F, Strauss M (2019). InsP6 binding to PIKK kinases revealed by the cryo-EM structure of an SMG1–SMG8–SMG9 complex. Nat Struct Mol Biol.

[17] Zhu L, Li L, Qi Y, Yu Z, Xu Y (2019). Cryo-EM structure of SMG1–SMG8–SMG9 complex. Cell Res.

[18] Alzahrani F, Kuwahara H, Long Y, Al-Owain M, Tohary M, AlSayed M, Mahnashi M, Fathi L, Alnemer M, Al-Hamed MH (2020). Recessive, deleterious variants in SMG8 expand the role of nonsense-mediated decay in developmental disorders in humans. Am J Hum Genet.

